# Dr. Charles Pinckney

**Published:** 1889-09

**Authors:** 


### DR. CHARLES PINCKNEY.

DR. CHARLES PINCKNEY died at his home in Atlanta on the 13th of last month. In his death the profession of this city loses a valued member. He descended from an illustrious family and to the inherited traits inseparable from such an ancestry were added the fruits of high intellectual training and fine culture. He was genial in disposition, faithful in friendship, refined and artistic in his tastes, and by his many admirable qualities of mind and heart secured the close friendship of those who knew him best. His professional attainments were of a high order and all who were so fortunate as to secure his services had implicit confidence in his judgment and skill. To his bereaved and sorrowing family the JOURNAL extends its sincere sympathy.

